# A Systematic Review of Lung Autopsy Findings in Elderly Patients after SARS-CoV-2 Infection

**DOI:** 10.3390/jcm12052070

**Published:** 2023-03-06

**Authors:** Susa Septimiu-Radu, Tejaswi Gadela, Doros Gabriela, Cristian Oancea, Ovidiu Rosca, Voichita Elena Lazureanu, Roxana Manuela Fericean, Felix Bratosin, Andreea Dumitrescu, Emil Robert Stoicescu, Iulia Bagiu, Mircea Murariu, Adelina Mavrea

**Affiliations:** 1 Department XIII, Discipline of Infectious Disease, “Victor Babes” University of Medicine and Pharmacy Timisoara, Eftimie Murgu Square 2, 300041 Timisoara, Romania; 2 Doctoral School, ‘’Victor Babes’’ University of Medicine and Pharmacy Timisoara, Eftimie Murgu Square 2, 300041 Timisoara, Romania; 3 School of General Medicine, Bhaskar Medical College, Amdapur Road 156-162, Hyderabad 500075, India; 4 Department of Pediatrics, Discipline of Infectious Disease, “Victor Babes” University of Medicine and Pharmacy Timisoara, Eftimie Murgu Square 2, 300041 Timisoara, Romania; 5 Center for Research and Innovation in Precision Medicine of Respiratory Diseases, “Victor Babes” University of Medicine and Pharmacy, Eftimie Murgu Square 2, 300041 Timisoara, Romania; 6 Cardioprevent Foundation, Calea Dorobantilor 3, Timisoara 300134, Romania; 7 Department of Radiology and Medical Imaging, “Victor Babes” University of Medicine and Pharmacy Timisoara, Eftimie Murgu Square 2, 300041 Timisoara, Romania; 8 Department of Microbiology, Multidisciplinary Research Center on Antimicrobial Resistance, “Victor Babes” University of Medicine and Pharmacy, Eftimie Murgu Square 2, 300041 Timisoara, Romania; 9 Department of Internal Medicine I, Cardiology Clinic, “Victor Babes” University of Medicine and Pharmacy Timisoara, Eftimie Murgu Square 2, 300041 Timisoara, Romania

**Keywords:** SARS-CoV-2, old age, COVID-19, elderly patients, autopsy, severe infection

## Abstract

Although COVID-19 may cause various and multiorgan diseases, few research studies have examined the postmortem pathological findings of SARS-CoV-2-infected individuals who died. Active autopsy results may be crucial for understanding how COVID-19 infection operates and preventing severe effects. In contrast to younger persons, however, the patient’s age, lifestyle, and concomitant comorbidities might alter the morpho-pathological aspects of the damaged lungs. Through a systematic analysis of the available literature until December 2022, we aimed to provide a thorough picture of the histopathological characteristics of the lungs in patients older than 70 years who died of COVID-19. A thorough search was conducted on three electronic databases (PubMed, Scopus, and Web of Science), including 18 studies and a total of 478 autopsies performed. It was observed that the average age of patients was 75.6 years, of which 65.4% were men. COPD was identified in an average of 16.7% of all patients. Autopsy findings indicated significantly heavier lungs, with an average weight of the right lung of 1103 g, while the left lung mass had an average weight of 848 g. Diffuse alveolar damage was a main finding in 67.2% of all autopsies, while pulmonary edema had a prevalence of between 50% and 70%. Thrombosis was also a significant finding, while some studies described focal and extensive pulmonary infarctions in 72.7% of elderly patients. Pneumonia and bronchopneumonia were observed, with a prevalence ranging from 47.6% to 89.5%. Other important findings described in less detail comprise hyaline membranes, the proliferation of pneumocytes and fibroblasts, extensive suppurative bronchopneumonic infiltrates, intra-alveolar edema, thickened alveolar septa, desquamation of pneumocytes, alveolar infiltrates, multinucleated giant cells, and intranuclear inclusion bodies. These findings should be corroborated with children’s and adults’ autopsies. Postmortem examination as a technique for studying the microscopic and macroscopic features of the lungs might lead to a better knowledge of COVID-19 pathogenesis, diagnosis, and treatment, hence enhancing elderly patient care.

## 1. Introduction

Coronavirus disease 2019 (COVID-19) is an extremely infectious viral illness caused by the SARS-CoV-2 virus that triggered a pandemic starting in March 2020 [1,2,3,4]. It is well-known from human research that COVID-19 is not only a respiratory illness but may evolve into a widespread disease affecting several organs and systems [5,6,7]. The association among SARS-CoV-2 and its receptor, the angiotensin-converting enzyme 2 (ACE2), might explain this behavior [8,9] since it is exhibited not just in the lung tissue, but also in cardiac cells, intestinal cells, hepatobiliary, and vascular endothelium, providing the virus possible accessibility to each of these organs [10,11,12,13].

Even though it is known that COVID-19 is a multi-systemic illness, the precise mechanism behind organ destruction continues to be uncertain. To comprehend the clinical signs, consequences, and pathophysiology of COVID-19, it is essential to examine the targets of SARS-CoV-2 replication and the viral spreading process [14,15]. The results of an autopsy may aid in elucidating the causes of disease processes and hence give essential information to guide treatment interventions [16,17]. However, the majority of published research has been on the clinical signs of COVID-19, its unique radiographic findings, and possible therapies [18,19,20]. A key disadvantage of small-scale autopsy investigations is their incapacity to definitively distinguish between COVID-19-induced injuries and unrelated diseases. This is further exacerbated by the fact that most patients who die from COVID-19 have numerous pre-existing comorbidities [21,22,23].

However, few autopsies may be a result of guidelines to postpone postmortems for people with potential or proven COVID-19 disease [24,25]. However, there are few corpses for examining COVID-19-related etiology and pathology, likely because performing autopsies on COVID-19 patients is very unsafe owing to the highly infectious tissue material, and it needs an autopsy facility with adequate biosecurity precautions [26]. Despite the fact that numerous review studies have emerged to describe various autopsy findings in adults or children, there is a lack of specificity with regard to specific autopsy findings in the elderly with COVID-19, who have the highest mortality rate among patients [27,28], and to a greater extent the aspect of the lungs in the elderly. Therefore, it is necessary to analyze the COVID-19 autopsy results in order to comprehend the COVID-related pathology in elderly individuals. This systematic review aims to describe the existing literature and studies on the observations involving the lung tissue described in autopsies of elderly patients who succumbed after SARS-CoV-2 infection.

## 2. Materials and Methods

### 2.1. Review Protocol

This systematic review was undertaken in December 2022 using three Internet databases: PubMed, Web of Science, and Scopus. It included literature published through December 2022. The following keywords were covered in this investigation: “COVID-19” OR “SARS-CoV-2” AND “autopsy” OR “necropsy” AND “elderly” OR “old” OR “elder” OR “senior”. The search was limited to English-language journal publications.

Using a structured and methodical search strategy in accordance with the Preferred Reporting Items for Systematic reviews and Meta-Analyses (PRISMA) [29] criteria and the International Prospective Register of Systematic Reviews (PROSPERO) guidelines [30], all relevant scientific papers investigating the autopsy findings of the lungs in the elderly were included in the analysis. The present systematic review was registered on the Open-Science Framework (OSF) platform [31]. The purpose of this systematic review was to address the following questions:

- *What are the most common autopsy features among elderly patients after COVID-19?*
- *Are there any anatomical particularities associated with SARS-CoV-2 mortality?*

### 2.2. Selection Process

The articles’ text, tables, figures, and other web resources served as the primary sources for all of the material that was acquired. The removal of duplicate submissions was the first step in the selection process, followed by the screening of the abstract and, finally, the screening of the complete text. The reference lists of the papers that were collected were carefully checked for material that was pertinent. Inclusion criteria comprised the following: (1) studies discussing the topic of mortality in elderly individuals with SARS-CoV-2 infection; (2) these studies must have reported autopsy findings only from patients whose deaths were attributed to COVID-19; (3) these studies must have described the involvement of the lungs. The exclusion criteria comprised: (1) the average age of the study population was under 70 years old, (2) a lack of data reporting risk variables, (3) studies that included patients with a cause of death different from SARS-CoV-2 infection, and (4) case reports, reviews, meta-analyses, letters to the editors, and short communications were also excluded. In the context of our review, we considered the following variables to be considered for reporting in this review: study number, first author’s name, country of the study, study year, study design, quality assessment, number of autopsies, mean or median age of the patients, patient’s sex, comorbidities, lung weight, pathologic findings on autopsy, and other interesting findings.

### 2.3. Data Extraction and Quality Assessment

**Figure 1:**
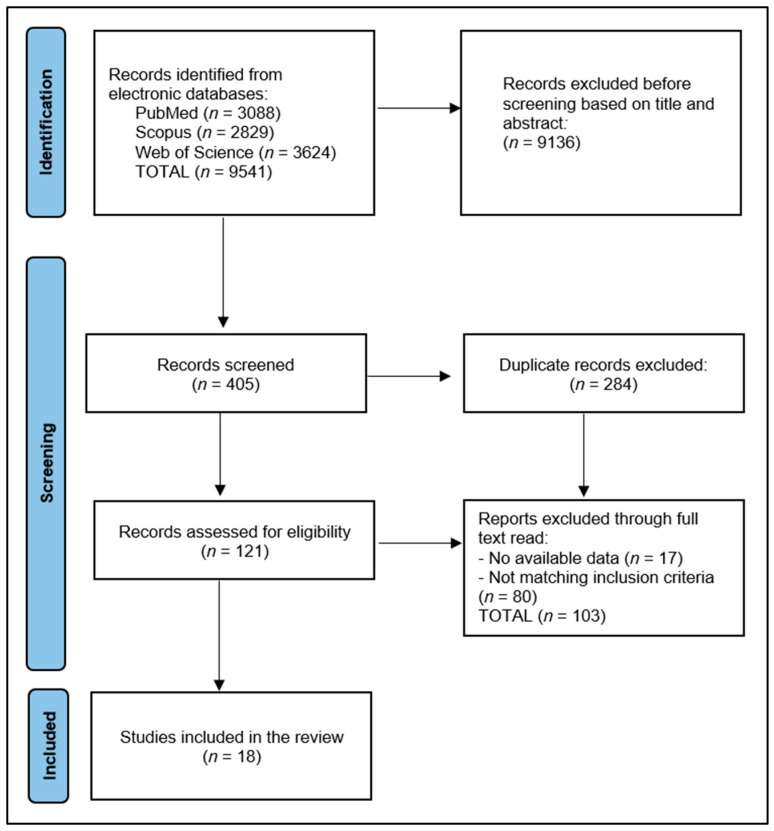
PRISMA flow diagram.

The initial results of the search revealed a total of 9541 articles, 284 of which were duplicates, as presented in Figure 1. After excluding 9136 papers based on their abstracts, we analyzed 121 full-text articles, and from those, we chose 18 to include in our systematic review. Using the Study Quality Assessment Tools offered by the National Heart, Lung, and Blood Institute (NHLBI), two researchers independently examined material from published papers and reported their findings. The tools are specific to study designs and may detect any methodological or operational issues. In the remaining research [32], the Quality Assessment Tool for Observational Cohort and Cross-Sectional Investigations was used. “Yes” answers were worth 1 point for each of a tool’s questions, while “No” and “Other” responses were worth 0 points. The final score for the performance was then computed. Therefore, studies with a score between 0 and 4 were regarded to be of fair quality, those with a score between 5 and 9 were assessed to be of good quality, and those with a score of 10 or more were deemed to be of excellent quality. To overcome the inherent biases of the included publications, two investigators were assigned to assess the quality of the selected research, therefore reducing the risk of selection bias, missing data, and measurement bias.

## 3. Results

A total of 478 autopsies were analyzed in the current systematic review on a total of 478 unique patients who died after acute severe SARS-CoV-2 infection confirmed through an RT-PCR test [33,34,35,36,37,38,39,40,41,42,43,44,45,46,47,48,49,50]. The majority of studies (13) were performed in Europe, while the other 4 were from the USA [36,41,43,47] and, respectively, 1 from China [38]. Out of the 18 autopsy studies, 13 were prospective studies performed with a thorough methodology. Ten studies were rated excellent by NHLBI standards, as seen in Table 1. 

Table 2 presents the summary of findings among the 478 autopsies from the included studies. The average age of patients was 75.6 years, with the highest mean of 82.9 years Skok et al. [45]. The majority of patients who were analyzed for autopsy were men, with an average of 65.4% over the included studies, the highest percentage being reported by Menter et al., with 80.9% male patients [34], respectively, the lowest percentage being reported by Youd and Moore [44], who indicated 44.4% male subjects. Regarding the reported comorbidities, COPD was identified in an average of 16.7% of all patients who died from severe SARS-CoV-2 infection, with the highest prevalence (44.4%) described by Youd and Moore [44]. The smoking status of the patients was scarcely described in only three studies [34,38,47]. 

The pathology findings of the lungs in elderly patients are described in Table 3. A total of 6 out of 18 studies reported the weight of the lungs, indicating that it was much higher than in normal healthy lungs [33,39,40,41,44,47]. The average weight of the right lung (RL) was 1103 g, while the left lung (LL) mass had an average of 848 g. Diffuse alveolar destruction (DAD) was described in a total of 67.2% of all deceased elderly patients with COVID-19. Pulmonary edema had a prevalence of 61.0% among the autopsies performed by Mikhaleva et al. [49], while Youd and Moore described pulmonary edema in 77.8% of all patients [44]. Thrombi formation was also described in the majority of studies, such as the Lax et al. study [33], where focal and extensive pulmonary infarctions were observed in 72.7% of patients, while Roden et al. described the same findings in 62.5% of the elderly patients who died. Pulmonary artery thrombosis was observed in all 40 patients analyzed by Skok et al. [45]. Deinhardt–Emmer et al. described thromboemboli in 50.0% of all patients [40]. 

Pneumonia and bronchopneumonia were also major findings among the deceased COVID-19 elderly patients. Menter et al. described this complication in 47.6% of the patients [34], while Grosse et al. reported the same complication in 78.6% of the 14 samples [39], and Skok et al. noted it in 89.5% of the 28 patients they analyzed [45]. Other autopsy findings of the lungs in the elderly patients who died from COVID-19 include the formation of hyaline membranes [33,35,36,38,40,46,47,48], the proliferation of pneumocytes and fibroblasts [33], extensive suppurative bronchopneumonic infiltrates, and an unevenly bluish–red color of the lung parenchyma [34], intra-alveolar edema and thickened alveolar septa [35], desquamation of pneumocytes, type II pneumocyte hyperplasia, alveolar infiltrates [36,38,42,46], multinucleated giant cell, and intranuclear inclusion bodies [36,37,42,47].

## 4. Discussion

### 4.1. Literature Findings

The findings of this systematic review provide crucial information on the pathological changes that occur in the lungs of older adults who succumb to COVID-19. Given that older adults are at higher risk of severe illness and death from COVID-19, understanding the specific changes that occur in their lungs can inform better treatment and management strategies for this vulnerable population. Second, the study’s focus on autopsy findings is particularly valuable, as autopsies can provide a detailed and comprehensive understanding of the macroscopic and microscopic pathological changes that occur in the lungs of COVID-19 patients. Autopsies are particularly important in cases where clinical data may not fully capture the extent of lung damage or may be confounded by other underlying conditions, as it was seen in this study that only a few patients suffered from COPD or other chronic lung disease. The findings of this systematic review can contribute to the growing body of literature on the long-term effects of COVID-19. While much attention has been given to the ARDS associated with severe COVID-19, less is known about the long-term pulmonary sequelae of the disease. Understanding the specific pathological changes that occur in the lungs of older adults with COVID-19 can inform ongoing research into the long-term effects of the disease and potentially guide the development of targeted interventions.

The engagement of the pulmonary system was one of the most prevalent occurrences as a result of SARS-CoV-2 infections. Several investigations demonstrated lung damage in dead individuals who had COVID-19 infection [51]. These individuals had been diagnosed with COVID-19. Diffuse alveolar destruction accompanied by the production of hyaline membranes is the primary histological finding in the lung, in addition to the presence of microthrombi in minor pulmonary arteries [52]. Despite the administration of a preventative dosage of an anticoagulant, it would seem that a significant prevalence of deep vein thrombosis exists, particularly among patients who are receiving critical care. Pulmonary embolism and risk for arterial thrombotic disease seem to be elevated in COVID-19 decedents, which suggests endothelial engagement in this process; however, further research is required [53].

Cytokine storm is a relatively new concept that has lately emerged as an important component of this illness. Patients who are afflicted by this disease display elevated concentrations of a number of critical cytokines, including IL-1 and IL-6, and a great number of others [54]. Interstitial pneumonia is made worse by these pro-inflammatory cytokines, which also contribute to the development of viral sepsis, which is characterized by substantial hypercoagulability [55]. These compounds may be studied in the autopsy as potential therapeutic options for some instances.

There has been an indication of extensive alveolar injury on the histological examination. Edema of the bronchial and alveolar epithelium, obstruction of the capillaries, and the creation of premature hyaline membranes lacking interstitial structure are the hallmarks of the earliest exudative stage of widespread alveolar injury, also known as diffuse alveolar damage [35]. Those who had to remain in the hospital for an extended period of time showed signs of both the proliferative and early fibrotic phases [56]. These stages are distinguished by the presence of alveolar thickening as well as desquamated type II pneumocytes in the alveoli [57]. These cells are defined by cytomegaly and enlarged nuclei, as well as vivid eosinophilic nucleoplasm. Nearly half of the inpatients had stroma, fibroblasts, and alveoli fibrin deposition, which is associated with the fibrinous and organizing phase of diffuse alveolar damage [42]. Extensive fibrosis, which is defined as the total loss of lung tissue, was identified in approximately 10% of the studied cases [43].

A minor proportion of the studied individuals had intra-alveolar neutrophilic invasion that was associated with bronchopneumonia and ranged from focal to generalized [39]. This might have been the consequence of inflammation or aspiration. It was observed that the majority of individuals had their pulmonary circulation investigated at autopsy. In fifty percent of the cases, a histopathological study of the pulmonary vessels revealed extensive thrombosis and thromboembolism in conjunction with microangiopathy [58]. Neutrophils were seen infiltrating the walls of blood vessels, which were linked with a hemorrhagic infarction of the lung parenchyma. It was shown that approximately two-thirds of individuals had fibrinoid necrosis of small arteries, making it a rather frequent observation [59].

Where immunohistochemistry was performed, it was observed that one-third of those patients were found to have inflammatory cell infiltrates [60]. These infiltrates included the existence of CD3+, CD4+, CD8+, and CD45+ lymphocytes, as well as CD68+ macrophages in the interstitium, alveoli, bronchi, and vasculature. Nevertheless, CD20+ B-lymphocytes were uncommon, and CD4+ and CD8+ lymphocytes showed a range of appearances, from being sparse in exudative alveolar damage to generating microscopic masses in individuals with fibroproliferative DAD in about 50% of the studied samples [61]. NK cells that were CD57+ were likewise in low supply. The appearance of CD61+ resident pulmonary megakaryocytes with nuclear hyperchromasia and atypia was the characteristic that stood out the most to the researchers. Platelet–fibrin complexes had formed in around 69% of the patients’ alveolar vasculature, which ultimately led to the development of thrombi [62]. Pneumocytes with a greater amount of RNA in the alveoli displayed clustering when stained with DNA.

An ultrastructural study revealed alterations in the tissue that were directly associated with the viral infection. In type I and type II pneumocytes, viral particles exhibited enclosed projections and a core that was surrounded by electron-dense granules on the periphery [63]. The nanoparticles were found inside cytoplasmic vacuoles as well as along plasmalemma membranes. The size of the particles was measured to be 82 nm, while the length of the projections was around 13 nm. Platelets and fibrin clots with incarcerated neutrophils were also seen in the lumina of alveolar vasculature in about two-thirds of cases [24]. 

On visual inspection, the lungs that were analyzed in the investigations that were included indicated that nearly half of the lungs were dense and congested, giving off a coloration that was similar to that of maroon tissue [64,65]. In more than half of the lungs, the parenchyma was found to be patchy to extensively edematous, and the texture was found to be solid but brittle [66,67]. On the exterior of the lungs that had been sliced, the individuals exhibited many bilateral pulmonary arterial clots, in addition to several regions of hematomas that were clearly evident [24]. In every single one of the studied lung samples, thrombosis of a medium or small-sized artery was discovered to be related to the infarction [33]. Approximately fifteen percent of the cases were reported to have a pulmonary embolism that was blocking the major pulmonary artery [68]. In addition, on visual examination, more than half of all cases exhibited focal to diffuse regions of consolidation with severe and widespread suppurative bronchopneumonic infiltrates [34]. 

There were a number of studies that documented the production of tiny thrombi in lung histology. In one investigation, there were fibrinous thrombi present in the alveolar arteries of 8 out of 10 individuals [62]. Additionally, different research found that over 90 percent of the patients had platelet–fibrin thrombi accumulated in the smaller pulmonary arteries [63]. Others have observed that during the autopsy, microscopic and/or macroscopic thrombi were present in 85% of the patients, and the most prevalent location where they were found was in the respiratory system [69]. There was a link between these results and the fact that the coagulation pattern of every patient was extended. The average PT time was 24 s, and the average activated partial thromboplastin time was 51 s.

On the other hand, COVID-19 is much less deadly in children, but there are studies reporting autopsy findings in the pediatric population that are similar to the adults and the elderly on various levels. It was observed that in the field of lung histopathology, widespread alveolar destruction was discovered in 78.3% of children’s autopsy specimens, which is comparable to the results of adult lung autopsies [70]. The results of the pathology of DAD caused by COVID-19 were comparable to those of different factors that trigger DAD [71]. There was a constant detection of fibrin infarcts in COVID-19, and it was described in practically all systems in pediatric autopsy in a number of investigations [72]. Several other results, including congestion, edema, and hemorrhage in a variety of organs, were described by a number of investigations [73]. In a retrospective study conducted by Yao et al. (2020), the authors analyzed autopsy reports from 26 patients who died from COVID-19 in China. They found that all 26 patients had diffuse alveolar damage, which is a pathological finding associated with ARDS [74]. Additionally, 23 of the 26 patients had pulmonary edema. The authors noted that the pulmonary edema in COVID-19 patients appeared to be more severe and widespread compared to pulmonary edema in patients with ARDS caused by other factors. In another study, the authors analyzed autopsy reports from 12 patients who died from COVID-19 in Germany [35]. They found that all 12 patients had diffuse alveolar damage and that 11 of the 12 patients had pulmonary edema. The authors noted that the pulmonary edema in COVID-19 patients appeared to be non-hydrostatic, meaning that it was not caused by heart failure or other non-pulmonary factors.

Nevertheless, the macroscopic and microscopic findings identified from lung autopsies should be corroborated with anatomical and molecular characteristics of patients affected by severe COVID-19. Among the hypothesized differences, it has been suggested that the hormone estrogen may have a protective effect against COVID-19 [75]. Estrogen has been shown to modulate the immune response and reduce inflammation, which are both important factors in the severity of COVID-19. Therefore, it is possible that female patients who died from severe COVID-19 may have less lung damage compared to male patients. Another hypothesis is that smoking has been shown to increase the risk of severe COVID-19 and is known to be more prevalent among men than women [76]. Smoking can also cause damage to the lungs, which may exacerbate lung damage in patients who contract COVID-19. Therefore, male patients who smoked may have more severe lung damage compared to female patients who did not smoke. In addition, there are recognized disparities in the immunological responses of men and women, with women usually possessing a higher immune response. It is probable that female patients who died from severe COVID-19 had a more powerful immune response, leading to a more efficient clearance of the virus and less lung damage than male patients. It is crucial to consider, however, that an excessively robust immune response may also cause tissue damage and may exacerbate COVID-19 symptoms [77].

### 4.2. Study Limitations

Even though the level of evidence in systematic reviews is high, there are several limitations that must be considered, such as the methods used to select and evaluate the included studies, as well as the potential conflicts of interest or funding sources. The inclusion criteria of the study may have introduced selection bias, specifically when the authors limited their search to studies published in English, which may have excluded relevant studies published in other languages. Additionally, the authors only included studies that reported lung autopsy findings, which may have excluded studies that reported on other aspects of COVID-19 pathology or treatment, as well as excluding other age groups that could serve as comparison of the reported findings. Another potential bias to consider is publication bias. Specifically, studies that report significant or novel findings are more likely to be published, while studies that report negative or null results may be less likely to be published. This may have influenced the selection of studies included in the systematic review and could have impacted the overall conclusions of the study. Overall, while this systematic review provides valuable insights into the lung pathology of elderly patients with COVID-19, it is important to consider the potential biases that may have influenced the selection and interpretation of the included studies. Future research in this area should strive to minimize these biases and increase transparency to ensure the reliability and validity of the findings.

## 5. Conclusions

Throughout the COVID-19 pandemic, numerous individuals from several demographic groups were infected with the SARS-CoV-2 virus. Yet, the group that was most severely affected by this pandemic was those who had already been identified with comorbidities, such as elderly patients; consequently, the majority of the deaths were elderly individuals with preexisting medical conditions. The majority of microscopic results could be linked to chronic diseases and old age, and the majority of the disease reported was universally applicable to the older population suffering from the most prevalent chronic diseases. The evidence presently available about COVID-19 infection-related fatality characterizes the systemic target of the infection as mostly the respiratory system, with patterns of severe damage in the elderly. The existence of many microthrombi in lung tissue shows that the pathogenesis of the virus includes some sort of coagulopathy; thus, future research should concentrate on demonstrating a solid relationship between the coagulation cycle and the pathophysiology of the SARS-CoV-2 virus. Some of the research included in this review may have incorrectly interpreted coincident findings as virus-dependent, given the high prevalence of comorbidities in the older group, and some of the studies may have disseminated skewed results. The impact of COVID-19 infection on the body’s natural defenses might be studied further in order to develop an early warning system and ways to prevent the infection from turning lethal, particularly in sensitive populations. From the perspective of autopsies, the problems of skilled staff, proper personal protective equipment, and facilities were the basis for conducting a thorough autopsy, which may differ across centers. Teamwork on a global scale is vital, and consistent diagnostic criteria are key considerations for any plan of action.

## Figures and Tables

**Table 1:** Study characteristics.

Study & Author	Country	Study Year	Study Design	Study Quality
1 [33] Lax et al.	Austria	2020	Prospective cohort	Excellent
2 [34] Menter et al.	Switzerland	2020	Retrospective cohort	Good
3 [35] Schaller et al.	Germany	2020	Prospective cohort	Fair
4 [36] Martines et al.	USA	2020	Retrospective cohort	Excellent
5 [37] Flikweert et al.	Netherlands	2020	Prospective cohort	Good
6 [38] Wu et al.	China	2020	Prospective cohort	Fair
7 [39] Grosse et al.	Austria	2020	Prospective cohort	Excellent
8 [40] Deinhardt–Emmer et al.	Germany	2021	Prospective cohort	Good
9 [41] Roden et al.	USA	2021	Retrospective cohort	Good
10 [42] Barisione et al.	Italy	2021	Prospective cohort	Excellent
11 [43] De Michele et al.	USA	2020	Prospective cohort	Excellent
12 [44] Youd and Moore	UK	2020	Retrospective cohort	Good
13 [45] Skok et al.	Austria	2021	Prospective cohort	Excellent
14 [46] Remmelink et al.	Belgium	2020	Retrospective cohort	Good
15 [47] Borczuk et al.	USA	2020	Prospective cohort	Excellent
16 [48] D’Onofrio et al.	Belgium	2022	Prospective cohort	Excellent
17 [49] Mikhaleva et al.	Russia	2021	Prospective cohort	Excellent
18 [50] Schwab et al.	Switzerland	2022	Prospective cohort	Excellent

**Table 2:** Summary of findings in the included studies.

Study Number	Autopsies	Age, Years (Mean/Median)	Sex (Men, %)	Lung Disease, Smoking
1 [33]	11	80.5	72.7%	COPD—18.2%
2 [34]	21	76.0	80.9%	COPD—18.2%; Smoking—14.3%
3 [35]	12	79.0	58.3%	COPD—16.7%; Lung cancer—8.3%
4 [36]	8	73.5	50.0%	COPD—25.0%
5 [37]	7	73.0	71.4%	NR
6 [38]	10	70.0	70.0%	COPD—20.0%; Smoking—50.0%
7 [39]	14	82.0	64.3%	COPD—42.9%
8 [40]	11	72.2	63.6%	COPD—18.2%
9 [41]	8	79.0	87.5%	COPD—12.5%
10 [42]	8	76.0	75.0%	None
11 [43]	40	71.5	70.0%	COPD—25.0%
12 [44]	9	72.1	44.4%	COPD—44.4%
13 [45]	28	82.9	60.7%	NR
14 [46]	17	71.0	70.6%	COPD—11.8%
15 [47]	68	73.0	69.0%	COPD—8.8%; Smoking—30.0%
16 [48]	44	82.0	56.8%	COPD—3.1%
17 [49]	100	70.5	47.0%	COPD—1%
18 [50]	62	76.5	65.0%	COPD—33.5%

NR—not reported; COPD—chronic obstructive pulmonary disease.

**Table 3:** Pathology findings of the lungs in the included studies.

Study Number	Lung Weight (Grams)	Pathologic Findings (%)	Other Findings
1 [33]	RL—998 g; LL—795 g	Massive bilateral congestion—81.8%; Moderate emphysematous changes—100%; Fibrous adhesions—63.6%; Focal or extensive pulmonary infarctions—72.7%	DAD, hyaline membranes, proliferation of pneumocytes and fibroblasts
2 [34]	NR	Severe mucous tracheitis/tracheobronchitis—30.0%; Heavy and firm lung parenchyma with severe congestion—100%; Proliferative DAD—38.1%; Bronchopneumonia—47.6%	Extensive suppurative bronchopneumonic infiltrates, unevenly bluish–red color, alveolar hemorrhage in conjunction with pulmonary embolism
3 [35]	NR	DAD—83.3%; Emphysema—16.7%; Minor neutrophil infiltration—41.7%	Hyaline membrane formation, intra-alveolar edema, and thickened alveolar septa
4 [36]	NR	DAD—87.5%; Squamous metaplasia and atypical pneumocytes—37.5%; Hemosiderin-laden macrophages and hemorrhage—50.0%; Interstitial pneumonitis—50.0%	Desquamation of pneumocytes, hyaline membranes, alveolar edema, and fibrin deposits, type II pneumocyte hyperplasia, alveolar infiltrates, increased alveolar macrophages
5 [37]	NR	Organized pneumonia—57.1%; DAD—14.7%; Intra-alveolar fibro myxoid/fibroblastic bodies—57.1%;	Micro-thrombi, hyperplasia with atypia, multinucleated giant cell, intranuclear inclusion bodies
6 [38]	NR	Fibrinous and suppurative alveolar exudation—90.0%;	Hyaline membrane and fibroblastic proliferation of alveolar septum, reactive hyperplasia and desquamation of the type II pneumocytes, acute bronchiolitis with mucous membrane exfoliation
7 [39]	RR—390 g to 1340 g; LL—350 g to 1100 g	DAD—100%; Type II pneumocyte hyperplasia—92.9%; Squamous metaplasia—92.9%; Multinucleated cells—85.7%; Secondary acute bronchopneumonia—78.6%	Pulmonary edema, significant bilateral vascular congestion, and dark red to grayish-red color of the lung parenchyma in the lower lobes and basal regions of the higher lobes
8 [40]	RR—1380 g; LL—1107 g	DAD—75.0%; Purulent bronchitis and bronchopneumonia—34.5%; Hyperemia and edema—75.0%; Thrombemboli—50.0%	Severe intra-alveolar and interstitial hemorrhages, severe loss of structured pneumocytes, hyaline membranes, fibrinous edema, and interstitial proliferation
9 [41]	RR+LL (average)—1220 g	Consolidation—62.5%; DAD—75.0%; Aspiration pneumonia—25.0%; Thrombemboli—62.5%; Perivascular chronic inflammation—50.0%	Hyaline membranes and interstitial fibroblast proliferation, interstitial fibroblast proliferation, organizing pneumonia
10 [42]	NR	Vascular injury and platelet microthrombi—50.0%; Early DAD—25.0%; Mid proliferative DAD—37.5%; Late DAD—37.5%	Type II pneumocyte hyperplasia, atypical pneumocytes, occasional multi-nucleation, and intracytoplasmic eosinophilic Mallory-like inclusions in type 2 pneumocytes
11 [43]	NR	Acute lung injury—72.5%; DAD—83.3%; Intravascular fibrin or platelet-rich aggregates—90.0%; Vascular congestion and hemangiomatosis-like changes—50.0%	Macroscopic and microscopic pulmonary thrombi
12 [44]	RR—932 g; LL—642 g	Lung consolidation—77.8%; Pulmonary edema—77.8%; Bronchopneumonia—33.3%	No macroscopic thromboemboli or areas of infarction
13 [45]	NR	Pulmonary artery thrombosis—100%; Bronchopneumonia—89.5%; Fibrosis—73.7%	Edema, proliferation, and hyaline membranes in all samples
14 [46]	NR	DAD—88.2%; Lung microthrombosis—64.7%; Bronchopneumonia—47.1%; Lung infarct—23.5%	Interstitial and alveolar fibroblastic proliferation, atypical pneumocytes, hyaline membranes, and type II pneumocyte hyperplasia
15 [47]	RR+LL (average)—>1300 g	Large vessel thrombi—42.0%; Focal and diffuse microthrombi—88.0%; DAD—16.2%; Type II pneumocytes hyperplasia—87.0%; Organizing pneumonia—34.0%	Hyaline membranes, atypical alveolar cells, basophilic intracytoplasmic inclusions
16 [48]	NR	DAD—84.1%; Fibrosis—59.1%	Fibrin deposition, hyaline membranes, atypic pneumocytes
17 [49]	NR	Pulmonary edema—61.0%; Hyaline membranes—67.0%; DAD—75.0%; Intra-alveolar hemorrhage—75.0%	Bronchial epithelium desquamation, alveolar macrophages, arterial thrombi, interstitial inflammation
18 [50]	NR	DAD—20.9%; Thrombosis—38.7%; Secondary pneumonia—27.4%	NR

NR—not reported; DAD—diffuse alveolar damage; RL—right lung; LL—left lung.

## Data Availability

Not applicable.
